# Prebiotic Activity of Vaginal Lactobacilli on Bifidobacteria: from Concept to Formulation

**DOI:** 10.1128/spectrum.02009-22

**Published:** 2023-01-05

**Authors:** Barbara Giordani, Angela Abruzzo, Carola Parolin, Claudio Foschi, Luca Laghi, Antonella Marangoni, Barbara Luppi, Beatrice Vitali

**Affiliations:** a Department of Pharmacy and Biotechnology, Alma Mater Studiorum, University of Bologna, Bologna, Italy; b Section of Microbiology, Department of Experimental, Diagnostic and Specialty Medicine, University of Bologna, Bologna, Italy; c Microbiology Unit, IRCCS Azienda Ospedaliero-Universitaria di Bologna, Bologna, Italy; d Department of Agricultural and Food Sciences, University of Bologna, Cesena, Italy; Universidade de Brasilia

**Keywords:** vaginal lactobacilli, prebiotics, bifidobacteria, cell-free supernatant, gut microbiota, hyaluronic acid gel

## Abstract

The gut of babies born vaginally is rapidly colonized by *Bifidobacterium* spp. after birth, while in infants born by cesarean section (C-section), the presence of bifidobacteria drops dramatically, increasing the risk of developing gastrointestinal disorders. Considering that newborns naturally come into contact with maternal lactobacilli as they pass through the birth canal, the aim of this work is to exploit for the first time the bifidogenic activity exerted by the cell-free supernatants (CFSs) from lactobacilli of vaginal origin, belonging to the species Lactobacillus crispatus, Lactobacillus gasseri, Limosilactobacillus vaginalis, and Lactiplantibacillus plantarum. CFSs were recovered after 7 h, 13 h, and 24 h of fermentation and assessed for the ability to stimulate the planktonic growth and biofilms of *Bifidobacterium* strains belonging to species widely represented in the gut tract. A bifidogenic effect was observed for all CFSs; such activity was maximal for CFSs recovered in exponential phase and was strongly dependent on the species of lactobacilli. Importantly, no stimulating effects on an intestinal Escherichia coli strain were observed. CFSs from L. vaginalis BC17 showed the best bifidogenic profile since they increased bifidobacterial planktonic growth by up to 432% and biofilm formation by up to 289%. The CFS at 7 h from BC17 was successfully formulated with a hyaluronic acid-based hydrogel aimed at preventing and treating breast sores in lactating women and exerting bifidogenic activity in infants born mainly by C-section.

**IMPORTANCE** Bifidobacteria in the gut tract of infants play crucial roles in the prevention of gastrointestinal diseases and the maturation of the immune system. Consequently, strategies to trigger a bifidogenic shift in the infant gut are highly desirable. Evidences suggest that the presence of a maternal vaginal microbiota dominated by health-promoting lactobacilli and the development of a bifidobacterium-enriched gut microbiota in newborns are interconnected. In this context, we found out that the cell-free supernatants from lactobacilli of vaginal origin were able to effectively stimulate the proliferation of *Bifidobacterium* spp. grown in free-floating and biofilm forms. The cell-free supernatant from *Limosilactobacillus vaginalis* BC17 showed excellent bifidogenic behavior, which was preserved even after its incorporation into a nipple formulation for lactating women. Lactobacilli derivatives, such as cell-free supernatants, have gained increasing interest by virtue of their safer profile than that of living cells and can be proposed as an ecosustainable approach to favor gut colonization of infants by bifidobacteria.

## INTRODUCTION

The human gut is colonized by more than 1,000 different species of bacteria, which greatly impact the physiology, immunology, and nutrition of the host (1). Although the intrauterine environment is commonly considered sterile, immediately after birth initial intestinal colonization occurs. The infant gut microbiota is characterized by less complexity than that of adults, in terms of both the total number of bacteria and the diversity of microbial taxa (2, 3). However, several factors may influence the establishment of the human intestinal microbiota, including the infant’s genetic signature, the diet, the intake of probiotics, the use of antibiotics during the perinatal period, as well as the nature of the delivery process. Recent studies have reported that normal colonization is most likely to occur when an infant is born full term by vaginal delivery and is exclusively breastfed during the first 6 months of life (4, 5). In babies born vaginally, the microorganisms found in the gut are similar to those comprising the maternal vaginal microbiota, with a prevalence of *Lactobacillus* spp. and *Bifidobacterium* spp. (6, 7). However, no difference in the prevalence or abundance of *Lactobacillus* between vaginally delivered and cesarean section (C-section)-delivered babies was observed in another study (8).

Among the members of the genus *Bifidobacterium*, species such as B. breve, B. bifidum, B. longum subsp. *infantis*, and B. longum subsp. *longum* have been associated with the development of a healthy infant gut microbiota, which is pivotal to preventing the onset of intestinal infections (9) and promoting the correct maturation of the immune system (5). On the contrary, in C-section-delivered infants, the main colonizers of the gut are bacteria present on the skin and in the hospital environment, such as Staphylococcus spp., Streptococcus spp., and propionibacteria (10), while the presence of bifidobacteria is dramatically reduced. Although these differences gradually decrease, a bacterial fingerprint remains associated with newborns delivered by C-section until 12 to 24 months of age, leading to a high risk for developing not only gastrointestinal disorders (i.e., diarrhea, irritable bowel syndrome, and colic) (11) but also other conditions such as allergies, obesity, colorectal cancer, cardiovascular disease, and type 2 diabetes (12, 13). Certainly, supplementation with bifidobacteria through the intake of probiotic preparations has positive effects on the infant (10). Another proposed way to increase bifidobacterial levels is the consumption of prebiotics, defined as the substrates that are selectively utilized by host microorganisms conferring a health benefit (14).

Considering that vaginally delivered newborns come into contact naturally with maternal lactobacilli as they pass through the birth canal, the aim of this work is to explore for the first time the bifidogenic activity of vaginal lactobacilli, assuming their potential use as prebiotics in early childhood. Lactobacilli are the dominant bacteria in the vaginal cavity of healthy women of reproductive age and play a crucial role in counteracting the onset of vaginosis, inflammatory states, and sexually transmitted infections (15). Furthermore, they are among the most studied probiotics and, when properly formulated, can be consumed orally to exert a beneficial action in the intestine (16). Thus, they could represent an intriguing alternative to widely utilized stimulants, consisting mostly of sugars such as inulin, arabinoxylans, galactooligosaccharides, and fructooligosaccharides (17). Recently, derivatives of probiotics, namely, postbiotics, have also gained interest due to their safer profile and better storage stability than those of living cells (18). Postbiotics are defined as soluble products or metabolites (e.g., cell-free supernatants [CFSs]) secreted by probiotics that are capable of providing physiological benefits to the target host (19). In the present study, we assessed the ability of the CFSs derived from 17 lactobacilli of vaginal origin to stimulate the *in vitro* proliferation of a panel of *Bifidobacterium* spp. typically present in the gut microbiota (B. breve, B. bifidum, B. longum subsp. *infantis*, B. longum subsp. *longum*, B. adolescentis, and B. angulatum). The aim was to identify a promising CFS to be included in a topical formulation for the prevention and treatment of breast sores in lactating women and the promotion of a favorable intestinal microbiota in the infant. In particular, hyaluronic acid (HA) was selected for the development of a nipple formulation by virtue of its hydrating and anti-inflammatory properties and the ability to favor tissue regeneration (20, 21). Here, we demonstrated that the CFS from Limosilactobacillus vaginalis BC17, when incorporated into a hyaluronic acid-based hydrogel, preserved its stimulating effect on planktonic cultures and biofilms of bifidobacteria.

## RESULTS

### Lactobacilli CFSs stimulate the planktonic growth of bifidobacteria.

The cell-free supernatants (CFSs) harvested from 17 lactobacilli strains of vaginal origin were assessed for their effects on the planktonic growth of 11 *Bifidobacterium* strains. The impact of CFSs on intestinal Escherichia coli SO107 was also investigated. CFSs were recovered at three time points, corresponding to the mid-exponential (7 h), late exponential (13 h), and stationary (24 h) growth phases in order to explore the dependence of the supernatant composition and the observed activity. The pH of lactobacilli CFSs was found to be slightly acidic after 7 h of incubation (pH 4.5 to 5.8), and it decreased as the incubation time increased (pH 4.1 to 5.4 for CFSs at 13 h and pH 3.7 to 4.5 for CFSs at 24 h). Considering that the optimum pH for the growth of bifidobacteria is 6.5 to 7.0 (22) and that the pH of the colon in newborns ranges between 5.5 and 7.5 (23, 24), the pH of CFSs was adjusted to 6.5 to mimic physiological conditions. Two batches of lactobacilli CFSs were tested against bifidobacteria and E. coli SO107, and good reproducibility between the data was found, as depicted in the correlation graphic in Fig. S1 in the supplemental material.

**FIG 1:**
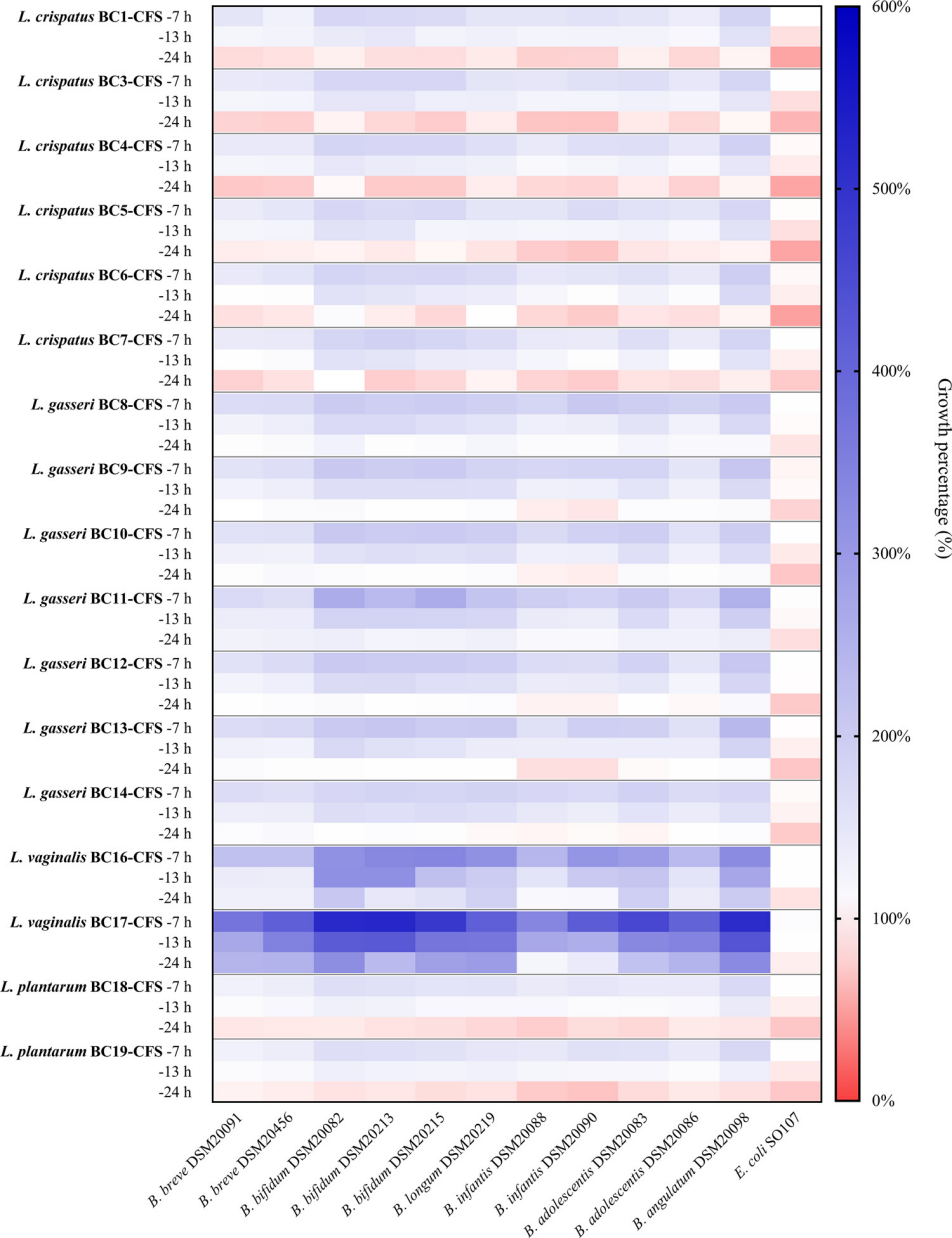
Effects of CFSs on *Bifidobacterium* species growth. The growth of bifidobacteria and E. coli SO107 in the presence of lactobacilli CFSs is reported as a percentage compared to the control (100%) (*n *= 5). The results obtained with CFSs recovered after 7 h, 13 h, and 24 h of fermentation are reported in the first, second, and third rows for each strain, respectively.

The heat map in Fig. 1 summarizes the overall results obtained for the effects of lactobacilli CFSs on bifidobacterial strains (numerical values are also reported in Table S1). The data clearly revealed that the growth of bifidobacteria differed when incubated in the presence of CFSs at 7 h, 13 h, and 24 h (*P < *0.01 by analysis of variance [ANOVA]). In addition, the effects exerted by each CFS varied depending on the *Bifidobacterium* strain.

For the short fermentation time of lactobacilli, a marked bifidogenic effect of CFSs was registered: the maximum bifidogenic effect was observed for CFSs at 7 h, which always allowed average bifidobacterial growth of at least 150%; all CFSs at 13 h significantly increased the average bifidobacterial growth, which ranged between 117.3% (BC18 CFS at 13 h) and 348.5% (BC17 CFS at 13 h). On the other hand, the average growth of *Bifidobacterium* spp. incubated with CFSs at 24 h ranged between 80.7% (BC3 CFS at 24 h) and 243.8% (BC17 CFS at 24 h), indicating that the results were strongly dependent on the CFS at 24 h considered. However, 14 out of 17 CFSs at 24 h failed to promote the proliferation of *Bifidobacterium* spp., and a significant stimulating effect was observed only for BC11 CFS at 24 h (average bifidobacterial growth of 124.4%), BC16 CFS at 24 h (average bifidobacterial growth of 156.1%), and BC17 CFS at 24 h.

The CFSs were also recovered from other species isolated from the vaginal mucosa, namely Enterococcus faecalis BC101, Enterococcus faecium BC105, Staphylococcus aureus SO105, and Staphylococcus epidermidis SO106. Interestingly, the CFSs (at 7 h, 13 h, and 24 h) recovered from these bacteria did not promote the growth of bifidobacteria (Fig. S2 and Table S2). Moreover, no increase in the growth of E. coli SO107 incubated with CFSs was observed (Fig. 1 and Fig. S2), suggesting that the stimulating effect on bifidobacteria was specifically exerted by lactobacilli CFSs. Moreover, all lactobacilli CFSs at 24 h significantly reduced E. coli SO107 growth by 8.6 to 46.7% (*P < *0.05 by a *t* test). Such an antibacterial effect was less pronounced for CFSs at 13 h since 9 out of 17 CFSs at 13 h only slightly decreased E. coli SO107 growth by 8.4 to 16.4% (*P < *0.05 by a *t* test), and it was completely absent for CFSs at 7 h.

**FIG 2:**
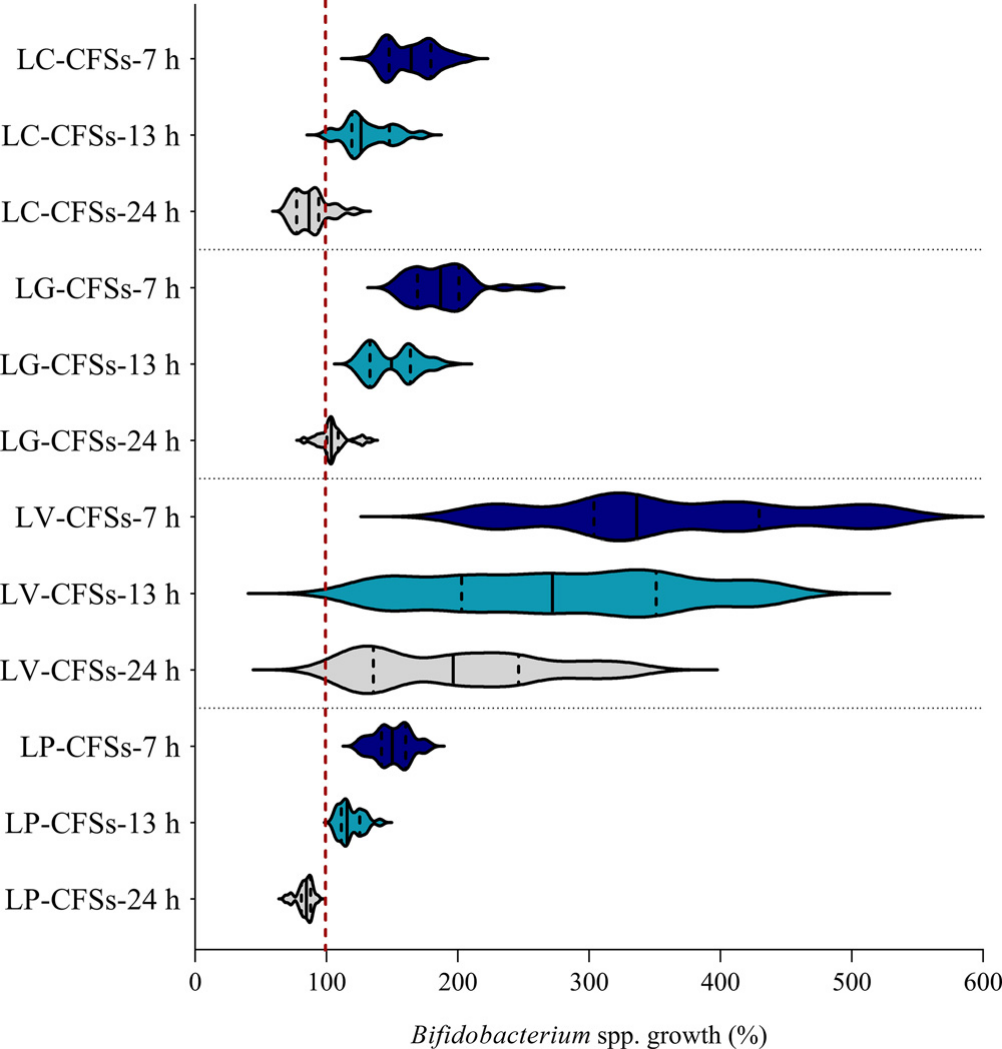
Effects of lactobacilli CFSs on *Bifidobacterium* species growth. Shown is a violin plot of the stimulating activities of L. crispatus (LC), L. gasseri (LG), *L. vaginalis* (LV), and *L. plantarum* (LP) CFSs at 7 h, 13 h, and 24 h on planktonic cultures of bifidobacteria. Solid and dotted lines represent the medians and quartiles, respectively.

The data reported in Fig. 1 were then grouped according to species (Lactobacillus crispatus, Lactobacillus gasseri, Limosilactobacillus vaginalis, and Lactiplantibacillus plantarum) in order to search for possible differences in bifidogenic activity among the CFSs from distinct lactobacilli species. The results are depicted in a violin plot in Fig. 2, showing the frequency distribution of the data of the activities of L. crispatus, L. gasseri, L. vaginalis, and L. plantarum CFSs towards all *Bifidobacterium* strains. Even considering one species at a time, the stimulating activity decreased as the fermentation time increased (*P < *0.01 by ANOVA). Furthermore, the activity of each lactobacilli species differed significantly from those of the other species (*P < *0.01 by ANOVA). The only exceptions were L. crispatus CFSs (at 7 h, 13 h, and 24 h) and L. plantarum CFSs (at 7 h, 13 h, and 24 h), which, on the contrary, exhibited very similar activities (*P > *0.05 by ANOVA), being the least effective in promoting bifidobacterial growth, as shown in Fig. 2. This is especially noticeable for CFSs at 24 h. Indeed, L. plantarum CFSs at 24 h significantly impaired the growth of all bifidobacteria (median residual growth of 84.8%), and L. crispatus CFSs at 24 h reduced the growth of at least 8 out of 11 *Bifidobacterium* strains (*P < *0.05 by a *t* test), with the exclusion of BC8 CFS at 24 h, which never hindered bifidobacterial growth. L. crispatus and L. plantarum CFSs at 13 h exhibited moderate prebiotic activity (median bifidobacterial growth of 126.3% and 115.7%, respectively), as did L. crispatus and L. plantarum CFSs at 7 h (median bifidobacterial growth of 164.6% and 150.2%, respectively). Particularly, BC8 CFS at 7 h and BC8 CFS at 13 h showed the highest bifidogenic activity among L. crispatus CFSs (at 7 h and 13 h) (*P < *0.01 by ANOVA).

L. gasseri CFSs at 7 h and L. gasseri CFSs at 13 h exerted a significant bifidogenic effect, leading to median bifidobacterial growth percentages of 186.9% and 149.5%, respectively (Fig. 2). The only strains of *Bifidobacterium* to be affected by L. gasseri CFSs at 24 h (except for BC11 CFS at 24 h) were B. longum subsp. *infantis* DSM20088 and DSM20090. In general, BC11 CFSs revealed the best bifidogenic profile among L. gasseri CFSs (at 7 h, 13 h, and 24 h) (*P < *0.01 by ANOVA).

By comparing all lactobacilli CFSs, BC17 CFSs were found to be the most effective in promoting the proliferation of bifidobacteria (*P < *0.01 by ANOVA), showing average bifidobacterial growth percentages of 442.0% (BC17 CFS at 7 h), 348.5% (BC17 CFS at 13 h), and 243.8% (BC17 CFS at 24 h).

### Lactobacilli CFSs stimulate the formation of bifidobacterial biofilms.

**FIG 3:**
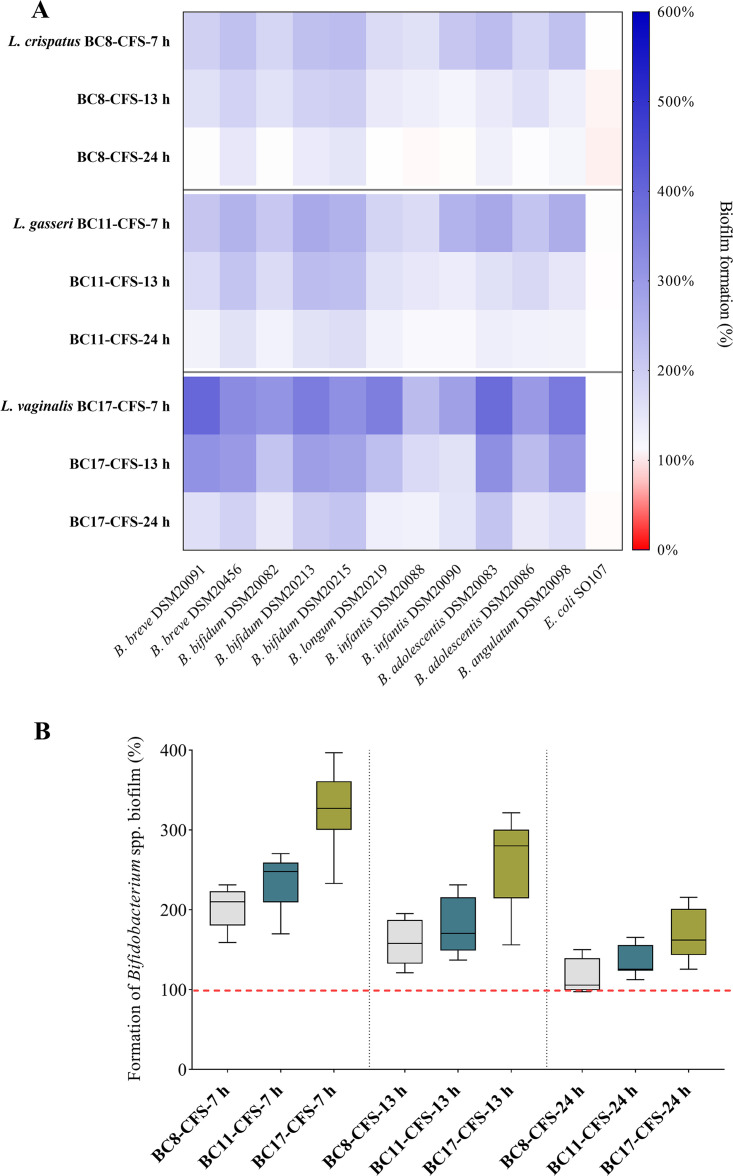
Effects of CFSs on *Bifidobacterium* species biofilms. (A) Formation of bifidobacterial and E. coli SO107 biofilms in the presence of BC8, BC11, and BC17 CFSs at 7 h, 13 h, and 24 h, reported as a percentage compared to the control (100%) (*n *= 3). (B) Box plot of the formation of bifidobacterial biofilms in the presence of BC8, BC11, and BC17 CFSs at 7 h, 13 h, and 24 h. Each box represents the interquartile range (25th to 75th percentiles). Lines within the boxes indicate the median values for the samples. The extremes of the bars indicate the minimum and maximum values, respectively.

Taking into account the results obtained on bifidobacterial planktonic growth, BC8 CFSs, BC11 CFSs, and BC17 CFSs were selected as models in order to further investigate the bifidogenic potential of L. crispatus, L. gasseri, and *L. vaginalis*, respectively. In this regard, the ability to promote the formation of biofilms is an important factor to guarantee stable gut colonization by bifidobacteria (25). The heat map in Fig. 3A illustrates the results for the activities of CFSs at 7 h, 13 h, and 24 h toward each strain of *Bifidobacterium* and E. coli SO107 (data are also reported in Table S3). In Fig. 3B, comparisons among BC8 CFSs, BC11 CFSs, and BC17 CFSs (at 7 h, 13 h, and 24 h) are depicted, considering the overall results obtained for each CFS.

Notably, BC8 CFSs, BC11 CFSs, and BC17 CFSs were able to stimulate not only the planktonic growth but also the biofilm formation of *Bifidobacterium* spp. Importantly, E. coli SO107 biofilm was not influenced by the presence of any of the lactobacilli CFSs evaluated.

Considering the overall results, the stimulation of biofilms varied significantly in the presence of CFSs at 7 h (average biofilm formation increase of 254.0%), CFSs at 13 h (195.9%), and CFSs at 24 h (139.2%) (*P < *0.01 by ANOVA), in agreement with what we observed previously for planktonic cultures of *Bifidobacterium* spp.

CFSs at 24 h recovered from BC11 and BC17 significantly promoted the formation of all bifidobacterial biofilms, with median percentages of 125.6% and 162.1%, respectively. Instead, BC8 CFS at 24 h appeared less efficient since it significantly stimulated the biofilm formation of only 5 out of 11 *Bifidobacterium* strains. The formation of bifidobacterial biofilms in the presence of CFSs at 13 h and 7 h was always greater than that of the control, although BC8 CFS was confirmed to be the least bifidogenic among the three CFSs considered (*P < *0.01 by ANOVA). Notably, BC17 CFSs displayed the greatest ability to enhance the formation of *Bifidobacterium* biofilms (254.9% for BC17 CFS at 13 h and 329.7% for BC17 CFS at 7 h), thus proving its excellent bifidogenic activity.

### Metabolomic analysis of BC17 CFSs.

Since BC17 CFSs revealed the best stimulating profile toward all *Bifidobacterium* spp. considered, their metabolomic profiles were investigated using ^1^H nuclear magnetic resonance (NMR), and the metabolite concentrations are reported in Table S4 as differences compared to the concentrations in sterile de Man-Rogosa-Sharpe (MRS) broth. The analysis allowed the identification of 55 molecules, mainly amino acids, organic acids, sugars, and alcohols, in agreement with what was observed in previous work (26, 27). Particularly, the fermentation of BC17 led to the production of alanine, aspartate, glutamate, isoleucine, leucine, proline, and valine, which are considered prebiotic amino acids (28). Moreover, aromatic amino acids (tryptophan and tyrosine), found mainly in BC17 CFSs at 7 h and 13 h, can be metabolized by *Bifidobacterium* species and converted into their lactic acid derivatives (29). Sulfur-containing amino acids are also required for the growth of bifidobacteria; indeed, some *Bifidobacterium* species (i.e., B. bifidum) have been reported to be cysteine auxotrophs, while others can synthesize cysteine from methionine (30). In this regard, the presence of methionine and cysteine in BC17 CFSs (with the latter being present mainly in BC17 CFS at 7 h) can sustain the proliferation of bifidobacteria. This stimulating activity can also be favored by the presence in BC17 CFS at 7 h of 4-hydroxyphenylacetate, a phenolic acid that is able to act as a modulator of the gut microbiota (31). Conversely, the concentrations of organic acids (acetate, formate, succinate, and lactate) and alcohols (ethanol and glycerol) that can potentially affect microbial growth were low in BC17 CFS at 7 h, while they increased with longer fermentation times (BC17 CFS at 13 h and BC17 CFS at 24 h).

### Characterization of BC17 CFS hydrogel.

Since the best bifidogenic profile was observed for BC17 CFS at 7 h, it was chosen for the preparation of a hyaluronic acid (HA)-based hydrogel. BC17 CFS at 7 h easily allowed the complete solubilization of HA, leading to the formation of a hydrogel characterized by a viscosity of 3,200 ± 200 mPa · s and a pH of 4.4 ± 0.5, not statistically different (*P > *0.05 by a *t* test) from those obtained by dissolving the HA in distilled water (viscosity equal to 3,000 ± 100 mPa · s and pH equal to 5.5 ± 0.8). No significant changes in the pH and viscosity were observed during the tested storage period compared to the values at time zero (*P > *0.05 by a *t* test).

**FIG 4:**
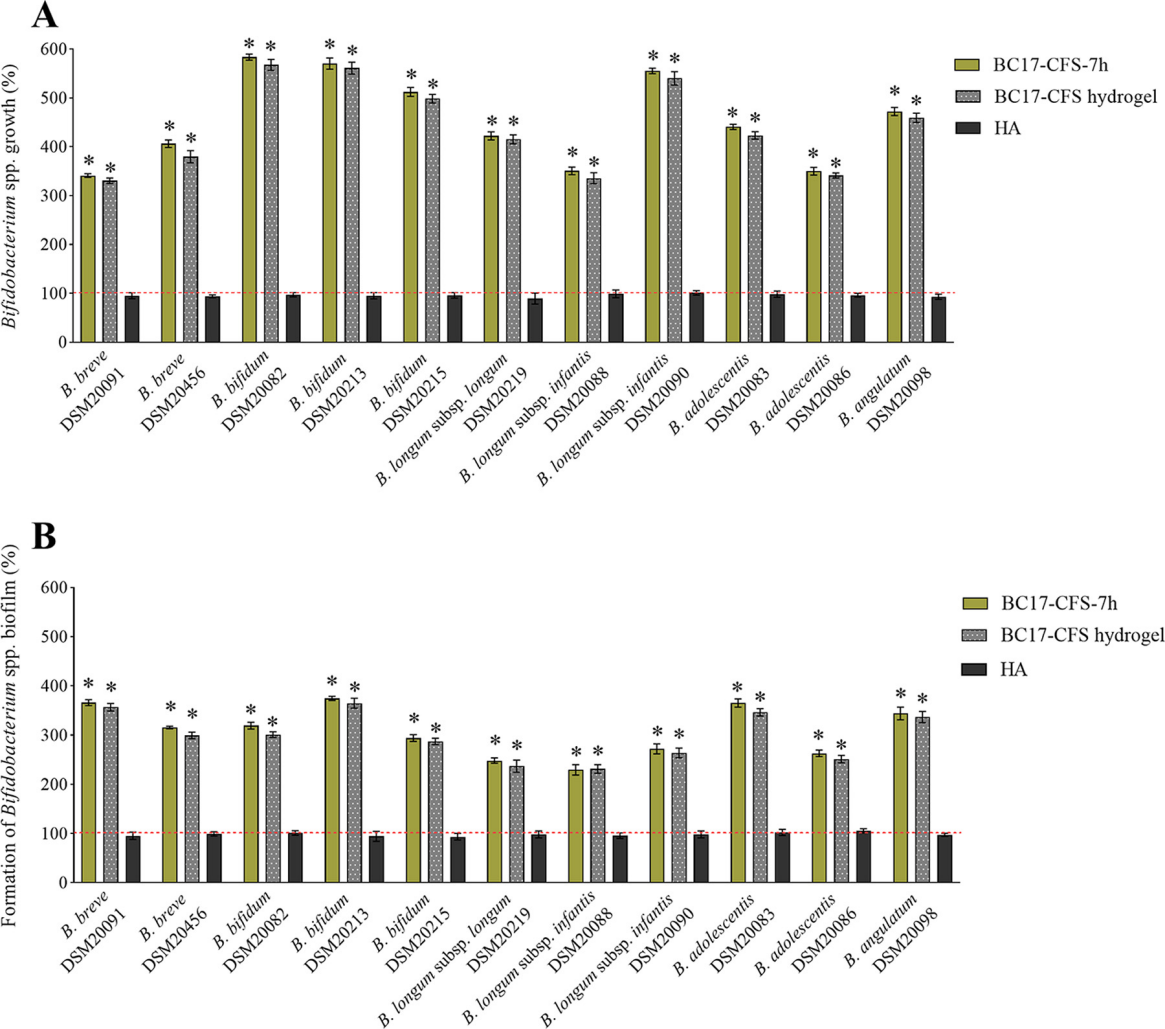
Bifidogenic activity of BC17 CFS hydrogel. The effects of BC17 CFS at 7 h, BC17 CFS hydrogel, and HA on bifidobacteria are expressed as percentages in terms of the stimulation of planktonic growth (A) and the stimulation of biofilm formation (B) (100%) (means ± SD [*n *= 4]). Statistical differences are calculated compared to the control (100%). *, *P < *0.05.

BC17 CFS hydrogel was then tested against *Bifidobacterium* spp. in order to investigate whether BC17 CFS at 7 h formulated with the HA-based hydrogel retained its beneficial effects on bifidobacterial planktonic growth and biofilm formation. The hydrogel was diluted 1/4 due to its high viscosity, and the results were compared to those obtained for BC17 CFS at 7 h and HA alone at the same dilution, as depicted in Fig. 4. The final pH of the diluted hydrogels was 6.50 ± 0.05, comparable to the pH of BC17 CFS at 7 h. It is worth noting that BC17 CFS at 7 h, diluted 1/4, stimulated the planktonic growth and biofilms of all bifidobacterial strains similarly to the more concentrated sample (*P > *0.05 by a *t* test), indicating that it was able to effectively preserve its bifidogenic properties even at a higher dilution.

HA did not affect the growth of *Bifidobacterium* spp. On the contrary, BC17 CFS hydrogel greatly increased the planktonic proliferation of all bifidobacterial strains considered (Fig. 4A), with growth percentages ranging from 330.7% (B. breve DSM20091) to 567.9% (B. bifidum DSM20082). Even bifidobacterial biofilms were positively stimulated by BC17 CFS hydrogel (Fig. 4B), with percentages of biofilm formation between 231.3% (B. longum subsp. *infantis* DSM20088) and 365.0% (B. bifidum DSM20215). Importantly, no significant differences between the activity of BC17 CFS hydrogel and that of BC17 CFS at 7 h were detected.

**FIG 5:**
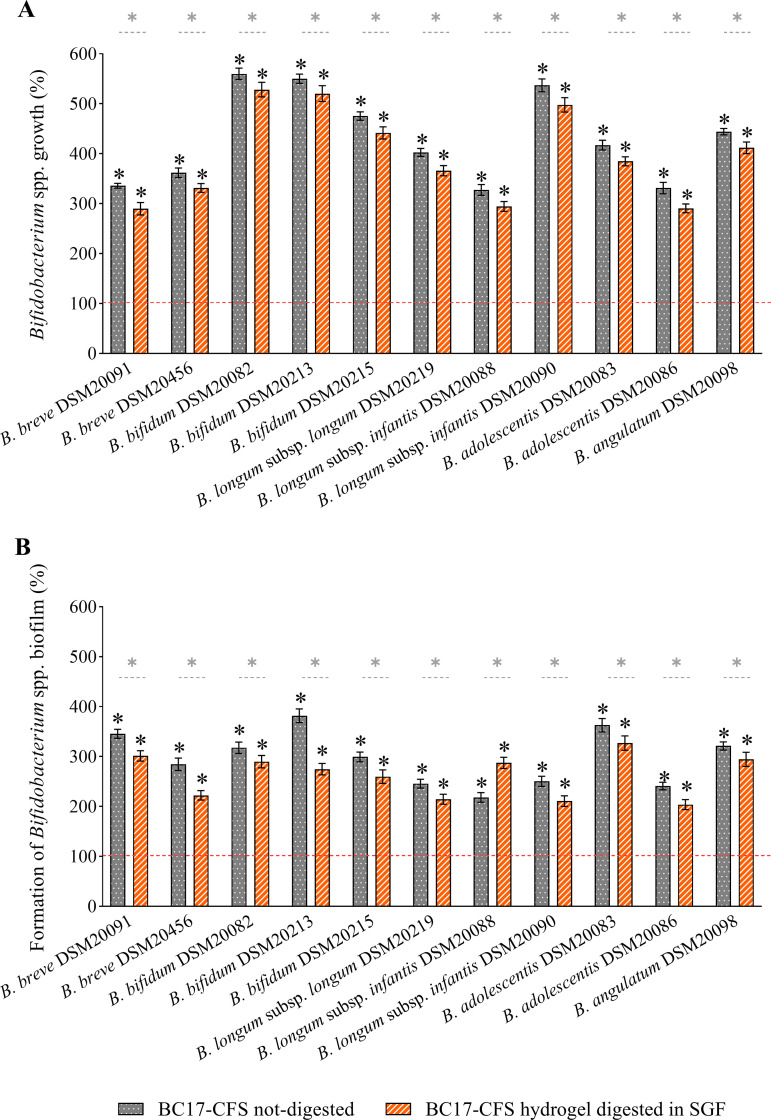
Bifidogenic activity of BC17 CFS hydrogel after digestion in simulated gastric fluid (SGF). The effects of BC17 CFS hydrogel not digested and digested in SGF are expressed as percentages in terms of the stimulation of planktonic growth (A) and the stimulation of biofilm formation (B) (100%) (means ± SD [*n *= 4]). Statistical differences compared to the control (100%) and between samples are reported. *, *P < *0.05.

Furthermore, the prebiotic activity of BC17 CFS hydrogel was preserved after 30 days of storage (*P > *0.05 by a *t* test) (Fig. S3). Finally, considering that the gastric half-emptying time in breastfed infants is 48 ± 15 min (24), BC17 CFS hydrogel was predigested in simulated gastric fluid (SGF) for 2 h to investigate the impact on prebiotic activity due to passage through the acidity of stomach. Although the activity decreased slightly compared to nondigested samples, BC17 CFS hydrogel was still able to significantly stimulate the free-floating bifidobacteria and their biofilms in the ranges of 290.5 to 528.3% (Fig. 5A) and 203.8 to 327.0% (Fig. 5B), respectively.

## DISCUSSION

The vaginal microbiota of reproductive-aged women is characterized by its relatively low complexity, being dominated by one Lactobacillus species among L. crispatus, L. gasseri, Lactobacillus jensenii, and Lactobacillus iners (32). It is well established that lactobacilli in the vaginal niche play an important role in the maintenance of a general state of health (33). In particular, the lactobacilli strains employed in the present study were derived from the vaginal mucosa of healthy premenopausal women and have been deeply characterized for their antagonistic effects on a plethora of pathogens responsible for vaginal and cervical infections, including *Candida* spp. (26, 27), Chlamydia trachomatis (34, 35), group B Streptococcus (36), and Neisseria gonorrhoeae (37). It has been observed that *Lactobacillus* spp. remain predominant even in the vaginal microbiota of healthy pregnant women, albeit both the alpha and beta diversities decrease (38, 39). On the contrary, a *Lactobacillus*-poor community during pregnancy as well as a high level of diversity within the vaginal microbiota are correlated with a high incidence of dysbiosis and an increased risk of preterm birth (40). In addition, in the first weeks of life of preterm infants, the level of gut colonization by *Bifidobacterium* spp. is considerably lower than that in full-term infants, similar to what occurs in babies born by C-section compared to those born naturally (41). This evidence suggests that the maternal vaginal microbiota and the development of the gut microbiota in newborns are somehow interconnected. While some beneficial properties of lactobacilli in the digestive tract of newborns are well characterized, such as the ability to increase intestinal immunity, protect from pathogens and inflammation, and enhance gut metabolic capacities (42–45), the direct effects on bifidobacteria are still poorly elucidated. In this context, the present study aimed to investigate whether lactobacilli isolated from the vaginal niche can positively affect the growth of bifidobacteria of intestinal origin, with a view to their potential use as prebiotics to generate a bifidogenic shift in the infant gut. Specifically, we first focused on the effects exerted by CFSs recovered after 7 h, 13 h, and 24 h of cultivation on the growth of free-floating bifidobacteria. Since the pH of lactobacilli CFSs decreased as the incubation time increased, probably due to the production of lactic acid and short-chain fatty acids (46), it was adjusted to 6.5 to mimic the physiological pH of the colon in newborns.

Our results highlighted that the stimulating effects on *Bifidobacterium* species growth were greater for lactobacilli CFSs recovered in exponential phase (Fig. 1). In particular, the highest bifidogenic activity was observed for CFSs at 7 h, which were all able to improve the proliferation of bifidobacteria by at least 50% compared to the growth of the control. The bifidogenic effects were less pronounced, but still evident, for CFSs at 13 h. Instead, only 3 out of 17 CFSs at 24 h retained the ability to sustain the proliferation of bifidobacteria, while the growth of most of them was slightly reduced in the presence of CFSs at 24 h from 8 lactobacilli. The inhibitory activity exerted by CFSs at 24 h could be attributable to the production of bacteriocin-like compounds, which are generally secreted by lactobacilli during the stationary phase of growth (47). On the other hand, the prevalent bifidogenic activity observed for CFSs at 7 h and 13 h may be due to the generation, at short incubation times, of partially degraded metabolites that serve as the substrates for other probiotics such as bifidobacteria. In this regard, Kang et al. discovered that a strain of Lactobacillus casei was able to produce a bifidogenic growth stimulator named 1,4-dihydroxy-2-naphthoic acid (48), but other studies are required to better clarify which metabolites and derivatives are ascribable to the bifidogenic activity. However, from our results, the bifidogenic activity is specific to lactobacilli since the CFSs from other bacteria isolated from the vaginal microenvironment (*Enterococcus* spp. and Staphylococcus spp.) did not exert any stimulating effects on *Bifidobacterium* species growth. Even more importantly, no growth-promoting effect on opportunistic gut pathogens such as E. coli was observed, suggesting that lactobacilli CFSs can actually act as prebiotics. It should also be highlighted that the functional properties of lactobacilli CFSs can vary among different species as a consequence of their diverse metabolic profiles. In the present study, we found that the CFSs from L. crispatus and *L. plantarum* were significantly less effective in promoting bifidobacterial proliferation than the CFSs from L. gasseri and *L. vaginalis* (Fig. 2). In particular, BC17 CFSs showed excellent bifidogenic properties at each recovery time (7 h, 13 h, and 24 h) and were selected, together with the most promising L. crispatus CFS (BC8 CFS) and L. gasseri CFS (BC11 CFS), for further investigation in terms of the ability to stimulate the formation of bifidobacterial biofilms. This aspect is particularly important considering that in natural environments the microbial biomass is found almost exclusively as a biofilm, which presents peculiar characteristics compared to its planktonic counterpart (25). Specifically, the ability of commensal *Bifidobacterium* spp. to form biofilms on the infant gut mucosa is pivotal for allowing stable colonization and blocking the adhesion of pathogens (49, 50). To the best of our knowledge, we proved for the first time that lactobacilli CFSs were able to promote not only the planktonic growth but also the formation of biofilms of *Bifidobacterium* spp. *in vitro*. The greatest stimulation of *Bifidobacterium* biofilms was achieved with CFSs at 7 h and CFSs at 13 h. Although other studies will be necessary to confirm the prebiotic activity of lactobacilli CFSs *in vivo*, our preliminary results allowed the selection of *L. vaginalis* BC17 as the best-performing sample (Fig. 3).

^1^H NMR analysis revealed that already after 7 h of fermentation, BC17 CFSs were rich in amino acids, which can promote the growth of bifidobacteria (see Table S4 in the supplemental material). Given the complex composition of lactobacilli CFSs, it is difficult to ascribe the prebiotic activity to a single molecule. Thus, the maximum bifidogenic effect was more likely to be obtained when stimulating molecules were already produced, while the presence of molecules with antimicrobial action (organic acids and alcohols) was still limited, as occurred in the CFSs recovered after 7 h of fermentation.

Finally, we proposed that BC17 CFS at 7 h can be included in a topical formulation aimed at breastfeeding mothers. Indeed, the development of a suitable formulation to be applied onto the nipples represents a crucial aspect of preventing and reducing wounds and damage and consequently limiting complications such as mastitis and breast abscess (51). The employment of a gel formulation is advantageous since it can create a moist environment at the application site, preventing wounds, decreasing local inflammation, and accelerating epithelial regrowth (52). Moreover, the rapid and simple application represents a beneficial approach, particularly when burning and stinging nipple pain occur (53). In this regard, the polymeric gel obtained through the direct solubilization of HA in BC17 CFS at 7 h had a pH in the range of that of the skin surface (pH 4.0 to 6.0) (54) and a viscosity suitable for achieving fast and easy application, thus covering the nipple and areola with a thin and homogeneous layer (55). Importantly, we proved that BC17 CFS hydrogel retained the bifidogenic potential of BC17 CFS at 7 h toward both planktonic cultures and biofilms of *Bifidobacterium* spp. (Fig. 4). Additionally, the technological (pH and viscosity) and functional (bifidogenic activity) (Fig. S3) properties of the hydrogel were maintained after 30 days of storage. The prebiotic activity was also almost preserved after digestion in simulated gastric fluid (Fig. 5), suggesting that the oral intake of the formulation by newborns during breastfeeding can effectively sustain bifidobacteria in the infant gut microbiota.

Since lactobacilli supernatants are side products of bacterial fermentation, their use as supplements for newborns offers some advantages, such as the low cost and ease of production, and deserves further investigation. With regard to future studies, the translation application of the present preliminary research could be verified by testing the bifidogenic effect of BC17 CFS hydrogel formulation in gut models inoculated with fecal slurries of infants born by C-section.

## MATERIALS AND METHODS

### Microorganisms and culture conditions.

The 17 lactobacillus strains employed in this study were isolated from vaginal samples of healthy premenopausal Caucasian women, according to the protocol approved by the Ethics Committee of the University of Bologna, Bologna, Italy (52/2014/U/Tess) (26). According to the recent reclassification of the *Lactobacillus* genus (56), these strains belong to the species Lactobacillus crispatus (BC1 to BC8), Lactobacillus gasseri (BC9 to BC14), *Limosilactobacillus vaginalis* (BC16 and BC17), and *Lactiplantibacillus plantarum* (BC18 and BC19). *Bifidobacterium* strains (B. breve DSM20091, B. breve DSM20456, B. bifidum DSM20082, B. bifidum DSM20213, B. bifidum DSM20215, B. longum subsp. *longum* DSM20219, B. longum subsp. *infantis* DSM20088, B. longum subsp. *infantis* DSM20090, B. adolescentis DSM20083, B. adolescentis DSM20086, and B. angulatum DSM20098) were purchased from the DSMZ (Braunschweig, Germany).

Lactobacilli and bifidobacteria were routinely cultured in de Man-Rogosa-Sharpe (MRS) broth (Difco, Detroit, MI, USA) supplemented with 0.05% l-cysteine (Merck, Milan, Italy) at 37°C in anaerobic jars containing Gas-Pak EZ (Beckton, Dickinson and Co., Milan, Italy).

Enterococcus faecalis BC101 and Enterococcus faecium BC105 belong to the Department of Pharmacy and Biotechnology of the University of Bologna. Staphylococcus aureus SO105, Staphylococcus epidermidis SO106, and Escherichia coli SO107 were isolated at Sant’Orsola-Malpighi University Hospital of Bologna, Italy, during routine diagnostic procedures. BC101, BC105, SO105, and SO106 are vaginal isolates, whereas SO107 is a human gut isolate. *Enterococcus* spp., Staphylococcus spp., and E. coli were grown aerobically at 37°C in nutrient agar (Difco).

### Preparation of CFSs from lactobacilli.

For each *Lactobacillus*/*Limosilactobacillus*/*Lactiplantibacillus* strain, 500 μL of a culture grown overnight (10^8^ CFU/mL) was used to inoculate 10 mL of MRS broth, and strains were allowed to grow for different times (7 h, 13 h, and 24 h). E. faecalis BC101, E. faecium BC105, S. aureus SO105, and S. epidermidis SO106, preventively grown on nutrient agar plates, were inoculated into MRS broth and subcultured as described above for lactobacilli.

At the end of each incubation, the supernatant was harvested by centrifugation (10,000 × *g* for 10 min) (Centrisart G-16C; Sartorius, Göttingen, Germany) and filtered through a 0.22-μm-pore-size filter (polyethersulfone [PES] 0.22-μm syringe filters; VWR International, Milan, Italy) to obtain CFSs at 7 h, 13 h, and 24 h. The final pH (pH50; Vio Lab Geass, Turin, Italy) was adjusted to 6.5 with 5 M NaOH (Merck), and samples were stored at −20°C until their use.

### Effects of lactobacilli CFSs on the planktonic growth of *Bifidobacterium* spp.

The prebiotic effects of CFSs derived from lactobacilli (two batches for each CFS) and control species (*Enterococcus* spp. and Staphylococcus spp.) on 11 *Bifidobacterium* strains (listed above) and E. coli SO107 were assessed. Bifidobacteria and E. coli were subcultured twice in MRS broth and then diluted in the same medium to reach a final concentration of 10^6^ CFU/mL. One hundred microliters of the bacterial suspension was inoculated into 96-multiwell round-bottom plates (Corning Inc., Pisa, Italy), and the same volume of CFSs was added. Positive-control wells contained 100 μL of the bacterial suspension and 100 μL of MRS broth. Wells filled with MRS broth only served as blank controls, while wells containing MRS broth and CFSs were included as sterility controls. Microplates were incubated under anaerobic conditions at 37°C, and growth was evaluated after 24 h of incubation by reading the absorbance at 600 nm (EnSpire multimode plate reader; PerkinElmer Inc., Waltham, MA, USA). Bifidobacterial and E. coli growth was calculated as a percentage relative to the absorbance of the corresponding positive controls.

### Effects of lactobacilli CFSs on biofilms of *Bifidobacterium* spp.

BC8, BC11, and BC17 were selected for the evaluation of the impact of lactobacilli CFSs on biofilms of bifidobacteria and E. coli, according to a protocol adapted from the one described previously by Parolin et al. (27). Bifidobacteria and E. coli were subcultured as described above, and cell pellets were resuspended in MRS broth to reach a final concentration of 10^7^ CFU/mL. CFSs at 7 h, 13 h, and 24 h derived from BC8, BC11, and BC17 were inoculated with the same amount of the bacterial suspensions into 96-multiwell round-bottom plates (200 μL per well), and biofilms were allowed to form under anaerobic conditions at 37°C for 48 h. Biofilms formed in the absence of CFSs were used as positive controls. At the end of the incubation period, the biofilm biomass was quantified by crystal violet staining (57). Briefly, the culture supernatant was discarded, and adherent cells were gently washed twice with sterile saline (0.9% [wt/vol] NaCl; Merck), fixed with 200 μL of absolute ethanol (Merck) for 5 min, and stained with 0.41% (wt/vol) crystal violet (Merck) for 5 min. After washing the wells with saline, the dye bound to adherent cells was resolubilized in 200 μL of ethanol, and the absorbance was measured at 595 nm. The stimulation of biofilm formation by the CFSs was expressed as a percentage relative to value for the control wells.

### ^1^H NMR analysis of BC17 CFSs.

The metabolomic analysis of BC17 CFSs was carried out as described previously by Parolin et al. (27) and as reported in the supplemental material.

### Preparation and characterization of BC17 CFS hydrogel.

*L. vaginalis* BC17 CFS at 7 h was selected for incorporation into a semisolid-dosage form, specifically, a hydrogel based on HA (molecular weight [MW], 1,800 to 2,300 kDa; Farmalabor SRL, Canosa di Puglia, Italy). HA was dissolved (2.5% [wt/wt]), with stirring (300 rpm), in the freshly prepared BC17 CFS at 7 h at room temperature for 24 h. A control sample was obtained by dissolving the same quantity of the polymer in ultrapure water (18.2 MΩ · cm) (MilliQ apparatus; Millipore, Milford, MA, USA). Immediately after preparation, BC17 CFS hydrogel was characterized in terms of pH (MicroPH Crison 2000; Modena, Italy) and viscosity using a rotational viscometer (23°C to 25°C, spindle TR11, 200 rpm, Visco Star; Fungilab SA, Barcelona, Spain). BC17 CFS hydrogel was stored under dark conditions at 4°C to 8°C, and the pH and viscosity were measured at fixed time intervals (7, 14, and 30 days).

The resulting BC17 CFS hydrogel was diluted 1/4 in distilled water and tested (immediately after preparation and after 30 days of storage) for the ability to stimulate the planktonic growth and biofilm formation of bifidobacteria, as described above.

To mimic passage through the acidic stomach, BC17 CFS hydrogel was diluted 1/4 in simulated gastric fluid (SGF) (125 mmol/L NaCl, 7 mmol/L KCl, 45 mmol/L NaHCO_3_, 3 g/L pepsin [pH 3]) and incubated for 2 h at 37°C (58); afterward, the pH was adjusted to 6.5, and the effects on the planktonic cultures and biofilms of bifidobacteria were assessed.

All analyses were performed on two preparations of BC17 CFS hydrogel, obtained from two batches of BC17 CFS at 7 h.

### Statistical analysis.

At least three independent assays were performed for each experiment (*n *= 3), and the results are expressed as means ± standard deviations (SD). Student’s *t* test was applied for the comparison of two means, and one-way ANOVA followed by Tukey’s correction was performed for multiple comparisons using GraphPad Prism version 9.2.0 for Windows (GraphPad Software, San Diego, CA, USA [www.graphpad.com]). Differences were deemed significant at a *P* value of <0.05.
