# Supplementary material for: Prebiotic Activity of Vaginal Lactobacilli on Bifidobacteria: from Concept to Formulation
Source: Microbiol Spectr. 2023 Jan 5;11(1):e02009-22. doi: 10.1128/spectrum.02009-22 (PMC9927276; doi:10.1128/spectrum.02009-22)
Supplement: Supplemental file 1 — Supplemental material. Download spectrum.02009-22-s0001.pdf, PDF file, 0.6 MB [file spectrum.02009-22-s0001.pdf]

Supplementary methods

^{1}HNM analysis of BC17-CFSs

BC17-CFSs (350 µL) were added to 350 µL of bi-distilled water and to 200 µL of a D_2O solution of 3-(trimethylsilyl)-propionic-2,2,3,3-d4 acid sodium salt (TSP) 10 mM as a chemical-shift reference (δ -0.017). A pH of 7.00 ± 0.02 was granted by a 1 M phosphate buffer, while microbial proliferation was prevented by 10 µL of NaN_3 2 mM. After centrifugation (18,630 × *g* for 10 min at 4°C), an AVANCE III spectrometer (Bruker, Milan, Italy) was used to register ^{1}H-NMR spectra at a frequency of 600.13 MHz and 298 K. Broad signals from slowly tumbling molecules were reduced with a T2 filter of 400 echoes, separated by an echo time of 400 µs. Chenomx software (Chenomx Inc., Canada, version 8.3) was employed to perform the assignment of signals to compounds, by comparison with Chenomx's (version 10) and HMDB's (release 2) databases. TSP was employed as an internal standard for the absolute quantification of molecules. The concentration of each molecule was calculated from the area of one of its signals, measured by global spectra deconvolution, implemented in MestReNova software (Mestrelab research S.L. Santiago De Compostela (Spain), version 14.2.0-26256), by considering a limit of quantification (LOQ) of 5. This was done after applying a line broadening of 0.3 and a baseline adjustment by Whittaker Smoother procedure. Concentrations of metabolites were expressed as differences with respect to MRS medium.

FIG S1 Correlation graphic between the two datasets (batch A and batch B) obtained testing lactobacilli CFSs towards planktonic cultures of bifidobacteria/*E. coli* SO107 (n = number of independent experiments) (Pearson r = 0.9779, R^2=0.9564).

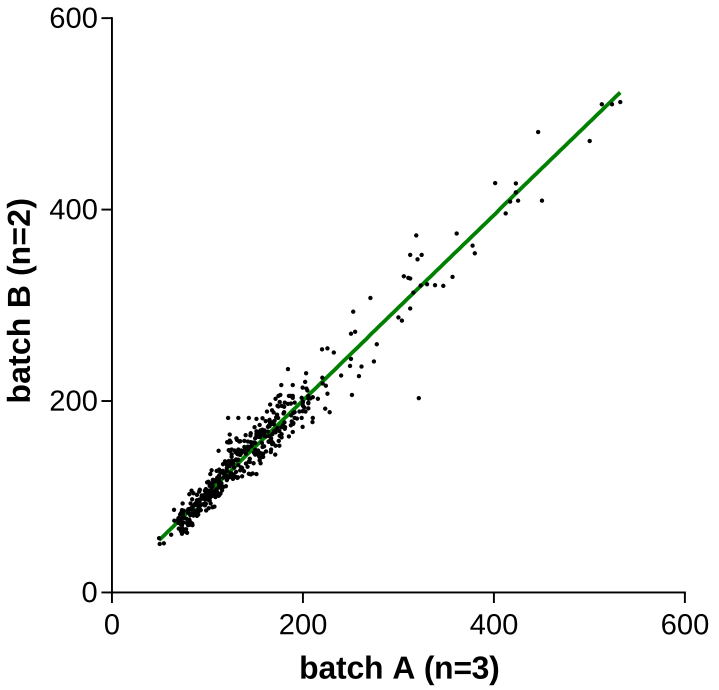

FIG S2 Effects of *Enterococcus*/*Staphylococcus* CFSs on bifidobacteria/*E. coli* SO107 growth. The growth of bifidobacteria/*E. coli* SO107 in the presence of CFSs is reported in percentage with respect to control (100%) (*n* = 3 from one batch of CFSs). Results obtained with CFSs recovered after 7 h, 13 h and 24 h of fermentation are reported in the first, second and third row of each strain, respectively.

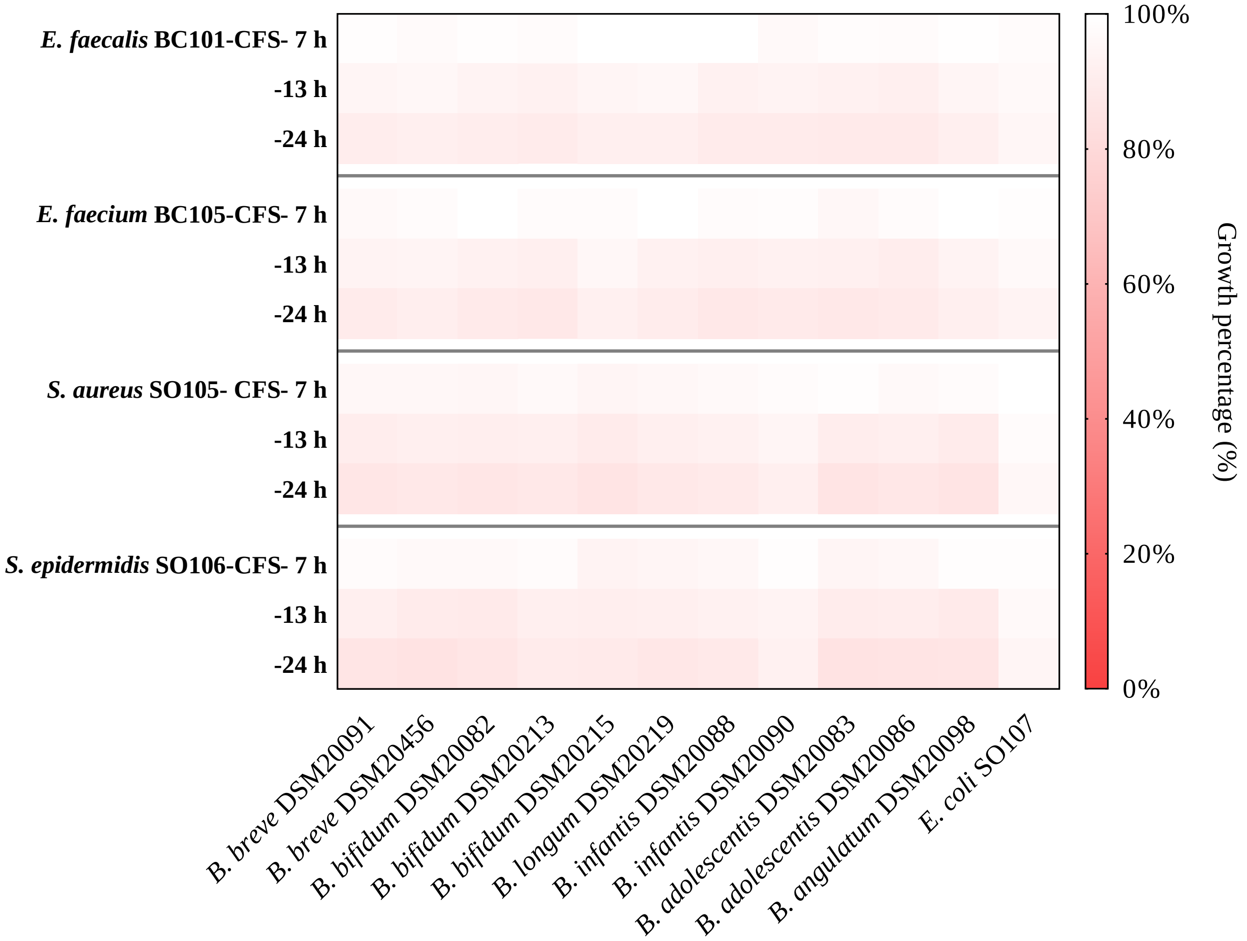

FIG S3 Effects of BC17-CFS-hydrogel on bifidobacteria tested immediately after preparation (t=0) and after 30 days of storage (t=30 days). Results are expressed in percentages in terms of (a) stimulation of planktonic growth and (b) stimulation of biofilms' formation (100%) (mean ± SD, n = 4). Statistical differences are calculated with respect to control (100%), * $p < 0.05$. Statistical differences between t=0 and t=30 days are also reported, ns: not significant.

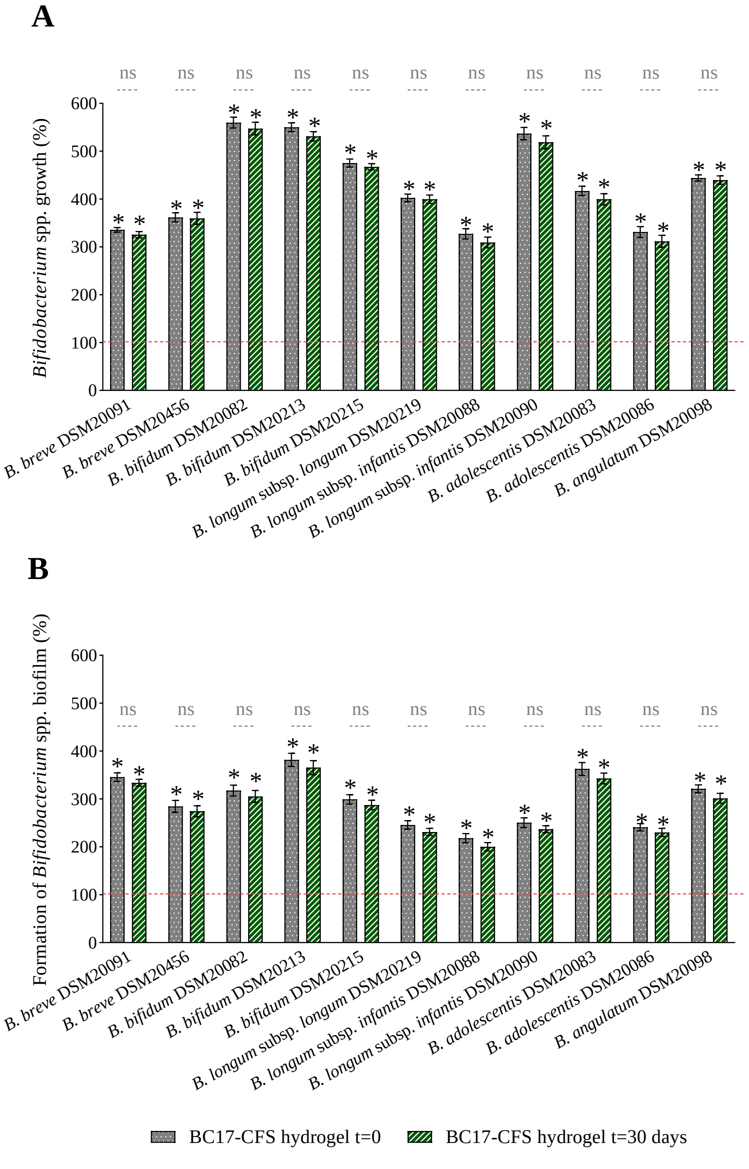

Table S1 Effects of lactobacilli CFSs on *Bifidobacterium* spp. growth. The growth of bifidobacteria/*E. coli* SO107 in the presence of lactobacilli CFSs is reported in percentage with respect to control (100%) as mean ± SD of data acquired from two batches of CFSs (*n* = 5).

	B. breve **DSM20091**	***B. breve*** **DSM20456**	***B. bifidum*** **DSM20082**	***B. bifidum*** **DSM20213**
L. crispatus **BC1-CFS** -7 h	149.24 ± 4.11	126.31 ± 20.65	178.30 ± 9.19	174.03 ± 8.04
-13 h	119.30 ± 2.52	122.55 ± 5.77	140.95 ± 6.23	146.59 ± 7.69
-24 h	81.30 ± 4.21	84.11 ± 3.40	91.90 ± 3.34	83.06 ± 4.80
L. crispatus **BC3-CFS** -7 h	142.29 ± 5.51	145.33 ± 7.51	177.04 ± 8.38	177.46 ± 7.02
-13 h	118.28 ± 4.47	120.60 ± 6.12	147.85 ± 5.85	147.91 ± 7.94
-24 h	76.64 ± 2.90	75.19 ± 3.39	93.70 ± 3.59	78.68 ± 7.46
L. crispatus **BC4-CFS** -7 h	141.21 ± 6.59	142.60 ± 5.92	183.68 ± 7.51	176.98 ± 7.27
-13 h	117.92 ± 4.75	120.85 ± 5.91	145.18 ± 6.07	138.13 ± 6.00
-24 h	70.98 ± 6.14	73.26 ± 3.22	96.48 ± 3.55	71.98 ± 3.20
L. crispatus **BC5-CFS** -7 h	137.83 ± 7.65	147.48 ± 6.83	176.50 ± 8.39	169.61 ± 6.35
-13 h	119.30 ± 7.11	120.04 ± 6.29	156.38 ± 8.55	152.49 ± 6.66
-24 h	90.44 ± 5.25	91.17 ± 3.40	93.68 ± 4.40	88.10 ± 3.91
L. crispatus **BC6-CFS** -7 h	142.57 ± 5.61	152.57 ± 6.21	182.78 ± 7.53	175.55 ± 7.28
-13 h	101.48 ± 4.06	103.62 ± 4.40	157.40 ± 8.71	149.81 ± 5.79
-24 h	83.62 ± 4.03	87.16 ± 4.29	106.90 ± 7.51	89.94 ± 12.25
L. crispatus **BC7-CFS** -7 h	140.06 ± 7.26	141.83 ± 7.38	181.86 ± 7.87	188.49 ± 17.93
-13 h	99.34 ± 6.86	107.66 ± 4.95	158.10 ± 7.53	149.51 ± 5.78
-24 h	76.11 ± 5.77	83.82 ± 4.40	99.68 ± 3.60	74.19 ± 3.04
L. gasseri **BC8-CFS** -7 h	167.32 ± 7.77	170.41 ± 8.54	200.62 ± 7.28	193.48 ± 8.55
-13 h	124.54 ± 5.11	132.99 ± 11.41	170.84 ± 7.41	170.77 ± 9.75
-24 h	102.48 ± 3.90	108.98 ± 5.96	123.74 ± 4.71	102.32 ± 6.69
L. gasseri **BC9-CFS** -7 h	154.54 ± 8.20	163.45 ± 17.47	207.28 ± 8.04	197.34 ± 10.70
-13 h	123.56 ± 5.44	131.52 ± 7.24	163.65 ± 9.15	162.77 ± 9.43
-24 h	99.90 ± 5.09	107.78 ± 6.80	108.40 ± 4.61	100.56 ± 4.90
L. gasseri **BC10-CFS** -7 h	156.97 ± 7.58	159.73 ± 7.92	207.48 ± 8.87	198.12 ± 8.16
-13 h	128.85 ± 7.21	125.74 ± 5.11	158.57 ± 12.14	164.23 ± 6.37
-24 h	103.47 ± 4.42	110.70 ± 6.12	106.70 ± 5.56	103.46 ± 4.00
L. gasseri **BC11-CFS** -7 h	171.36 ± 10.21	164.33 ± 11.50	261.87 ± 13.32	233.41 ± 26.33
-13 h	133.78 ± 6.88	137.19 ± 8.18	184.34 ± 9.97	184.66 ± 8.88
-24 h	124.27 ± 6.79	128.46 ± 9.49	131.73 ± 6.92	120.18 ± 5.93
L. gasseri **BC12-CFS** -7 h	158.19 ± 6.81	169.45 ± 9.16	205.44 ± 10.89	198.22 ± 8.82
-13 h	122.65 ± 4.65	130.27 ± 8.12	169.87 ± 6.65	172.21 ± 7.33
-24 h	101.26 ± 4.98	104.28 ± 4.03	108.82 ± 4.17	101.64 ± 4.40
L. gasseri **BC13-CFS** -7 h	168.54 ± 7.65	172.94 ± 9.25	205.64 ± 7.64	210.82 ± 19.09
-13 h	127.41 ± 8.43	123.65 ± 4.93	172.61 ± 8.07	159.61 ± 12.43
-24 h	106.94 ± 9.62	102.50 ± 4.05	103.46 ± 4.78	101.04 ± 4.00
L. gasseri **BC14-CFS** -7 h	167.10 ± 7.17	161.76 ± 6.97	177.51 ± 7.97	185.13 ± 14.46
-13 h	131.68 ± 6.17	134.04 ± 5.90	165.25 ± 6.63	162.09 ± 6.26
-24 h	105.85 ± 5.95	110.97 ± 4.08	100.48 ± 3.72	105.32 ± 4.28

***L. vaginalis* BC16-CFS** -7 h	219.92 ± 8.46	220.87 ± 8.96	314.86 ± 11.76	331.42 ± 19.78
-13 h	138.36 ± 7.08	135.08 ± 5.78	318.76 ± 14.41	317.94 ± 15.66
-24 h	129.33 ± 7.28	129.15 ± 4.69	209.42 ± 12.29	146.22 ± 33.65
***L. vaginalis* BC17-CFS** -7 h	371.81 ± 15.91	413.56 ± 15.44	518.34 ± 20.80	524.37 ± 22.99
-13 h	270.15 ± 13.96	345.95 ± 19.35	421.21 ± 15.90	424.89 ± 16.38
-24 h	244.48 ± 11.40	247.90 ± 9.51	322.49 ± 12.07	234.73 ± 11.66
***L. plantarum* BC18-CFS** -7 h	126.86 ± 8.09	133.56 ± 6.26	163.03 ± 7.24	159.93 ± 12.53
-13 h	107.46 ± 7.14	114.56 ± 4.21	129.37 ± 6.29	125.10 ± 4.82
-24 h	86.97 ± 5.90	88.47 ± 4.59	88.58 ± 4.29	85.34 ± 4.46
***L. plantarum* BC19-CFS** -7 h	126.23 ± 7.74	133.59 ± 5.30	163.67 ± 8.52	161.65 ± 7.12
-13 h	107.48 ± 6.20	111.98 ± 4.37	130.21 ± 6.34	123.67 ± 6.57
-24 h	93.19 ± 3.72	90.16 ± 3.64	84.52 ± 3.51	87.08 ± 4.77
	***B. bifidum* DSM20215**	***B. longum* DSM20219**	***B. infantis* DSM20088**	***B. infantis* DSM20090**
***L. crispatus* BC1-CFS** -7 h	170.53 ± 6.61	149.39 ± 8.76	150.19 ± 8.06	158.07 ± 9.72
-13 h	122.76 ± 4.82	127.46 ± 9.48	120.60 ± 7.14	121.90 ± 6.51
-24 h	82.38 ± 3.15	88.78 ± 3.72	75.39 ± 6.21	76.75 ± 6.42
***L. crispatus* BC3-CFS** -7 h	177.50 ± 7.33	149.43 ± 7.03	147.85 ± 5.45	160.75 ± 8.42
-13 h	130.08 ± 8.57	132.98 ± 15.26	119.76 ± 4.91	121.46 ± 7.61
-24 h	73.14 ± 7.05	89.90 ± 4.89	69.13 ± 5.44	68.73 ± 7.08
***L. crispatus* BC4-CFS** -7 h	176.66 ± 7.22	161.53 ± 6.91	142.23 ± 6.93	161.43 ± 7.13
-13 h	132.67 ± 5.08	128.67 ± 5.16	113.66 ± 4.72	119.34 ± 6.01
-24 h	72.32 ± 9.47	90.44 ± 5.51	78.76 ± 3.63	77.16 ± 6.56
***L. crispatus* BC5-CFS** -7 h	172.90 ± 6.14	149.33 ± 5.56	150.65 ± 6.88	168.65 ± 8.36
-13 h	121.28 ± 4.44	123.30 ± 5.87	118.86 ± 5.36	119.64 ± 7.55
-24 h	94.92 ± 4.75	85.62 ± 6.00	73.07 ± 5.32	69.31 ± 5.88
***L. crispatus* BC6-CFS** -7 h	177.06 ± 7.86	170.41 ± 8.58	145.31 ± 6.29	149.81 ± 9.05
-13 h	139.25 ± 10.43	136.55 ± 10.33	116.42 ± 6.86	103.06 ± 3.86
-24 h	79.24 ± 3.94	99.06 ± 4.01	78.61 ± 6.44	72.45 ± 3.09
***L. crispatus* BC7-CFS** -7 h	182.16 ± 7.39	168.35 ± 8.09	142.77 ± 7.17	140.57 ± 16.14
-13 h	139.19 ± 8.48	136.87 ± 8.61	118.98 ± 8.35	102.76 ± 3.92
-24 h	79.43 ± 6.11	93.58 ± 7.92	77.32 ± 4.81	73.02 ± 2.74
***L. gasseri* BC8-CFS** -7 h	198.84 ± 7.13	191.44 ± 8.70	181.92 ± 7.49	207.82 ± 8.54
-13 h	160.85 ± 6.83	151.47 ± 7.21	130.59 ± 7.65	133.34 ± 13.99
-24 h	106.12 ± 6.10	119.18 ± 4.61	105.98 ± 9.31	106.24 ± 7.86
***L. gasseri* BC9-CFS** -7 h	202.58 ± 8.36	184.94 ± 9.88	178.13 ± 17.97	183.83 ± 18.30
-13 h	162.83 ± 8.70	161.33 ± 9.40	128.48 ± 9.72	129.74 ± 7.35
-24 h	101.32 ± 5.26	104.92 ± 6.02	89.58 ± 8.02	86.80 ± 6.63
***L. gasseri* BC10-CFS** -7 h	202.90 ± 8.58	195.02 ± 10.23	171.95 ± 7.71	189.48 ± 9.86
-13 h	160.43 ± 6.57	163.77 ± 7.78	130.98 ± 7.66	131.93 ± 10.19
-24 h	103.80 ± 5.09	106.58 ± 3.91	92.26 ± 5.10	89.74 ± 4.56
***L. gasseri* BC11-CFS** -7 h	261.29 ± 20.79	213.78 ± 16.16	195.58 ± 8.96	186.99 ± 18.30
-13 h	182.53 ± 12.77	176.93 ± 13.74	139.79 ± 7.79	133.71 ± 6.54
-24 h	123.46 ± 10.72	127.59 ± 7.94	113.70 ± 13.51	111.48 ± 11.97

***L. gasseri* BC12-CFS** -7 h	199.96 ± 7.46	193.44 ± 13.10	167.57 ± 14.61	166.65 ± 13.65
-13 h	162.39 ± 7.29	160.25 ± 7.12	137.44 ± 18.66	140.23 ± 23.22
-24 h	102.72 ± 5.96	105.06 ± 5.36	92.80 ± 10.67	92.72 ± 13.00
***L. gasseri* BC13-CFS** -7 h	199.28 ± 17.02	200.46 ± 16.70	159.93 ± 11.51	193.24 ± 22.53
-13 h	152.71 ± 11.73	138.29 ± 13.56	135.14 ± 13.97	136.37 ± 19.39
-24 h	101.20 ± 5.97	101.28 ± 4.04	83.00 ± 3.48	82.48 ± 3.25
***L. gasseri* BC14-CFS** -7 h	182.07 ± 13.79	187.84 ± 8.35	176.93 ± 12.08	171.08 ± 9.31
-13 h	168.37 ± 11.66	161.27 ± 6.04	143.33 ± 14.21	133.59 ± 5.20
-24 h	103.62 ± 4.45	96.14 ± 4.66	94.52 ± 7.88	97.08 ± 8.48
***L. vaginalis* BC16-CFS** -7 h	336.59 ± 19.76	315.58 ± 17.78	239.79 ± 13.30	306.32 ± 14.40
-13 h	222.18 ± 8.85	199.90 ± 16.27	151.63 ± 7.76	204.02 ± 27.86
-24 h	155.87 ± 9.50	189.08 ± 16.09	113.04 ± 4.78	114.62 ± 5.26
***L. vaginalis* BC17-CFS** -7 h	488.92 ± 24.73	412.04 ± 20.28	335.90 ± 19.73	419.25 ± 18.04
-13 h	369.92 ± 20.15	366.91 ± 14.85	269.17 ± 24.18	258.47 ± 14.45
-24 h	285.75 ± 22.92	295.12 ± 12.54	118.86 ± 9.66	139.43 ± 9.97
***L. plantarum* BC18-CFS** -7 h	151.75 ± 10.60	153.99 ± 12.53	139.87 ± 7.72	148.55 ± 10.06
-13 h	114.14 ± 4.66	114.40 ± 6.57	115.40 ± 5.86	107.68 ± 6.68
-24 h	83.00 ± 3.63	79.42 ± 7.22	73.79 ± 11.88	81.75 ± 10.84
***L. plantarum* BC19-CFS** -7 h	156.79 ± 6.60	148.35 ± 5.55	143.93 ± 13.97	158.93 ± 13.93
-13 h	123.46 ± 5.32	125.66 ± 8.44	118.62 ± 8.07	116.82 ± 10.44
-24 h	81.68 ± 3.03	85.04 ± 3.93	72.13 ± 4.44	68.89 ± 3.25
	***B. adolescentis* DSM20083**	***B. adolescentis* DSM20086**	***B. angulatum* DSM20098**	***E. coli* SO107**
***L. crispatus* BC1-CFS** -7 h	150.81 ± 10.19	135.83 ± 11.80	185.96 ± 10.14	100.60 ± 4.00
-13 h	122.64 ± 6.19	113.94 ± 5.58	157.13 ± 9.76	84.60 ± 3.24
-24 h	92.10 ± 6.03	78.92 ± 5.56	94.18 ± 4.06	53.31 ± 2.70
***L. crispatus* BC3-CFS** -7 h	162.91 ± 12.51	146.79 ± 11.97	180.80 ± 13.63	101.50 ± 3.79
-13 h	126.24 ± 9.29	120.08 ± 8.63	148.11 ± 10.72	83.60 ± 3.21
-24 h	88.42 ± 4.07	78.57 ± 5.76	95.08 ± 4.56	61.61 ± 2.51
***L. crispatus* BC4-CFS** -7 h	163.75 ± 7.57	146.41 ± 7.44	189.28 ± 7.17	98.68 ± 6.26
-13 h	127.31 ± 5.28	113.68 ± 4.34	147.79 ± 6.42	88.78 ± 7.74
-24 h	89.08 ± 4.47	76.24 ± 4.27	94.06 ± 4.00	52.41 ± 4.39
***L. crispatus* BC5-CFS** -7 h	158.65 ± 10.49	149.99 ± 14.38	177.98 ± 8.23	98.94 ± 3.72
-13 h	125.80 ± 11.17	115.60 ± 5.34	156.85 ± 5.86	83.82 ± 4.69
-24 h	86.74 ± 3.34	90.36 ± 3.37	93.70 ± 5.41	53.83 ± 3.19
***L. crispatus* BC6-CFS** -7 h	159.59 ± 12.03	144.19 ± 8.90	194.58 ± 12.27	97.12 ± 3.68
-13 h	125.43 ± 5.27	106.12 ± 8.67	173.13 ± 16.01	91.48 ± 4.21
-24 h	86.26 ± 4.41	82.48 ± 3.35	94.00 ± 3.98	50.41 ± 1.91
***L. crispatus* BC7-CFS** -7 h	164.55 ± 6.94	140.07 ± 10.39	182.44 ± 9.75	102.64 ± 3.89
-13 h	126.27 ± 6.65	101.10 ± 3.78	153.19 ± 9.27	91.78 ± 4.06
-24 h	85.12 ± 3.74	83.20 ± 3.49	90.96 ± 5.66	72.81 ± 3.28
***L. gasseri* BC8-CFS** -7 h	195.94 ± 11.39	188.20 ± 11.71	205.86 ± 8.18	99.94 ± 4.22
-13 h	153.83 ± 12.87	126.26 ± 8.16	172.81 ± 7.43	97.86 ± 4.89
-24 h	120.54 ± 5.37	110.64 ± 7.59	111.88 ± 6.36	86.10 ± 4.16

***L. gasseri* BC9-CFS** -7 h	183.84 ± 10.81	150.42 ± 5.71	210.38 ± 10.76	95.78 ± 4.25
-13 h	151.35 ± 6.19	127.75 ± 9.81	171.62 ± 10.76	97.04 ± 5.34
-24 h	104.34 ± 4.71	104.02 ± 5.03	109.50 ± 6.93	77.80 ± 3.00
***L. gasseri* BC10-CFS** -7 h	194.32 ± 8.17	158.33 ± 10.80	197.36 ± 19.13	101.12 ± 3.77
-13 h	161.09 ± 7.32	129.44 ± 8.42	168.30 ± 11.05	90.02 ± 4.84
-24 h	108.82 ± 5.62	103.40 ± 3.86	108.16 ± 7.05	71.89 ± 8.08
***L. gasseri* BC11-CFS** -7 h	204.54 ± 7.79	176.87 ± 12.13	251.29 ± 16.83	104.08 ± 4.26
-13 h	170.69 ± 9.50	136.47 ± 12.15	195.02 ± 9.55	96.42 ± 4.73
-24 h	127.09 ± 7.79	126.80 ± 6.64	133.83 ± 8.12	82.46 ± 4.59
***L. gasseri* BC12-CFS** -7 h	186.78 ± 7.17	150.70 ± 6.89	208.02 ± 9.14	101.04 ± 4.33
-13 h	147.59 ± 12.66	121.54 ± 4.53	179.76 ± 6.92	98.44 ± 9.08
-24 h	99.08 ± 9.76	96.24 ± 8.14	112.44 ± 4.18	71.61 ± 3.88
***L. gasseri* BC13-CFS** -7 h	190.28 ± 7.30	159.07 ± 22.24	237.42 ± 18.24	99.22 ± 4.26
-13 h	136.85 ± 13.17	135.38 ± 20.34	184.80 ± 11.63	92.30 ± 3.45
-24 h	97.36 ± 3.63	101.72 ± 3.99	104.38 ± 3.93	71.27 ± 4.76
***L. gasseri* BC14-CFS** -7 h	189.16 ± 9.72	170.01 ± 8.01	176.54 ± 13.98	97.88 ± 3.69
-13 h	155.53 ± 6.66	138.87 ± 10.25	160.41 ± 16.10	93.60 ± 4.46
-24 h	94.55 ± 4.82	100.36 ± 10.44	103.94 ± 3.92	72.99 ± 2.73
***L. vaginalis* BC16-CFS** -7 h	295.88 ± 16.00	233.76 ± 20.42	327.03 ± 13.01	101.76 ± 3.79
-13 h	212.18 ± 23.46	152.53 ± 28.00	274.14 ± 65.85	102.04 ± 4.81
-24 h	192.44 ± 11.84	137.73 ± 19.88	200.46 ± 10.41	85.76 ± 4.04
***L. vaginalis* BC17-CFS** -7 h	460.37 ± 26.06	405.82 ± 17.68	512.04 ± 19.17	105.84 ± 4.30
-13 h	331.50 ± 16.31	340.66 ± 32.20	434.19 ± 27.86	101.92 ± 4.30
-24 h	218.55 ± 13.23	245.75 ± 20.32	328.68 ± 25.14	91.34 ± 4.43
***L. plantarum* BC18-CFS** -7 h	143.04 ± 9.35	142.59 ± 16.34	172.69 ± 6.86	101.32 ± 4.60
-13 h	109.55 ± 7.19	112.50 ± 6.27	140.61 ± 8.73	91.60 ± 3.41
-24 h	79.01 ± 8.30	88.66 ± 4.21	86.50 ± 5.41	71.05 ± 3.11
***L. plantarum* BC19-CFS** -7 h	158.11 ± 10.89	142.71 ± 17.53	176.02 ± 7.17	99.66 ± 4.44
-13 h	116.08 ± 6.27	107.42 ± 5.51	130.49 ± 9.69	89.10 ± 3.69
-24 h	81.46 ± 3.04	87.76 ± 6.16	83.44 ± 3.61	72.01 ± 2.72

Table S2 Effects of *Enterococcus*/*Staphylococcus* CFSs on bifidobacteria/*E. coli* SO107 growth. The growth of bifidobacteria/*E. coli* SO107 in the presence of CFSs is reported in percentage with respect to control (100%) as mean ± SD of data acquired from one batch of CFSs (n = 3).

	***B. breve* DSM20091**	***B. breve* DSM20456**	***B. bifidum* DSM20082**	***B. bifidum* DSM20213**
***E. faecalis* BC101-CFS**- 7 h	98.54 ± 2.56	97.21 ± 4.25	98.54 ± 5.21	97.44 ± 3.78
-13 h	94.5 ± 4.78	95.41 ± 5.78	93.22 ± 6.23	92.41 ± 4.56
-24 h	90.21 ± 3.25	91.24 ± 6.12	90.23 ± 5.14	89.43 ± 2.28
***E. faecium* BC105-CFS**- 7 h	96.54 ± 3.67	97.42 ± 3.78	100.01 ± 4.56	97.62 ± 3.36
-13 h	93.41 ± 3.51	94.14 ± 4.56	92.32 ± 3.98	91.51 ± 4.14
-24 h	89.43 ± 4.71	90.73 ± 2.23	88.52 ± 3.78	87.94 ± 5.21
***S. aureus* SO105- CFS**- 7 h	95.41 ± 5.36	95.62 ± 3.46	94.83 ± 4.56	96.52 ± 7.12
-13 h	90.4 ± 4.78	91.21 ± 2.56	90.74 ± 6.21	91.54 ± 3.38
-24 h	86.51 ± 6.32	87.54 ± 4.78	86.51 ± 4.12	87.81 ± 4.45
***S. epidermidis* SO106-CFS**- 7 h	97.52 ± 4.91	96.53 ± 6.13	96.51 ± 3.36	97.82 ± 5.47
-13 h	91.41 ± 5.12	89.51 ± 2.25	88.55 ± 3.95	91.52 ± 3.78
-24 h	86.34 ± 3.26	85.14 ± 3.36	86.42 ± 4.05	89.55 ± 4.12
	***B. bifidum* DSM20215**	***B. longum* DSM20219**	***B. infantis* DSM20088**	***B. infantis* DSM20090**
***E. faecalis* BC101-CFS**- 7 h	100.02 ± 6.21	100.04 ± 7.12	99.52 ± 8.13	96.52 ± 3.89
-13 h	94.54 ± 2.39	95.52 ± 4.52	92.21 ± 2.28	93.44 ± 7.05
-24 h	91.53 ± 5.17	91.43 ± 3.84	89.12 ± 3.45	89.53 ± 3.99
***E. faecium* BC105-CFS**- 7 h	97.55 ± 3.23	100.04 ± 4.45	97.44 ± 5.24	98.32 ± 6.2
-13 h	95.61 ± 5.62	92.31 ± 6.32	91.12 ± 3.38	92.33 ± 4.71
-24 h	92.05 ± 6.32	89.72 ± 4.65	87.53 ± 4.56	88.71 ± 6.64
***S. aureus* SO105- CFS**- 7 h	94.32 ± 2.48	95.63 ± 5.27	96.52 ± 6.78	97.84 ± 2.56
-13 h	89.54 ± 3.35	91.44 ± 5.84	92.51 ± 6.54	94.53 ± 3.32
-24 h	85.42 ± 4.23	87.65 ± 4.16	88.93 ± 3.84	91.51 ± 5.93
***S. epidermidis* SO106-CFS**- 7 h	93.61 ± 6.32	94.62 ± 5.32	95.82 ± 3.33	98.74 ± 4.75
-13 h	90.63 ± 7.14	91.64 ± 3.89	92.42 ± 5.26	93.64 ± 2.29
-24 h	88.62 ± 2.56	87.44 ± 2.37	88.45 ± 6.18	92.53 ± 3.84
	***B. adolescentis* DSM20083**	***B. adolescentis* DSM20086**	***B. angulatum* DSM20098**	***E. coli* SO107**
***E. faecalis* BC101-CFS**- 7 h	98.32 ± 3.79	97.84 ± 4.36	99.18 ± 6.28	97.77 ± 6.32
-13 h	92.41 ± 5.23	91.45 ± 3.34	94.46 ± 6.21	96.51 ± 4.71
-24 h	88.91 ± 4.25	88.92 ± 4.57	91.49 ± 4.78	94.93 ± 4.59
***E. faecium* BC105-CFS**- 7 h	95.43 ± 3.37	97.84 ± 5.68	99.51 ± 3.38	98.79 ± 5.89
-13 h	91.84 ± 4.65	90.11 ± 4.32	93.37 ± 6.51	96.41 ± 4.39
-24 h	87.52 ± 2.32	88.64 ± 2.33	91.39 ± 3.12	93.66 ± 5.27
***S. aureus* SO105- CFS**- 7 h	98.72 ± 4.39	96.47 ± 4.17	97.81 ± 4.18	99.76 ± 7.13
-13 h	90.51 ± 6.21	91.19 ± 3.27	89.52 ± 5.13	97.37 ± 3.98
-24 h	85.63 ± 3.74	87.09 ± 4.32	85.58 ± 4.56	95.52 ± 4.59
***S. epidermidis* SO106-CFS**- 7 h	94.54 ± 5.52	95.61 ± 5.41	98.67 ± 5.23	98.73 ± 3.56
-13 h	89.62 ± 3.99	90.11 ± 5.02	88.69 ± 6.21	96.47 ± 3.27
-24 h	85.12 ± 5.31	85.58 ± 2.39	86.28 ± 5.56	94.28 ± 4.78

Table S3 Effects of lactobacilli CFSs on *Bifidobacterium* spp. biofilms. The formation of bifidobacteria/*E. coli* SO107 biofilms in the presence of BC8-BC11-BC17 CFSs-7 h, CFSs-13 h and CFS-24 h is reported in percentage with respect to control (100%) as mean ± SD of data acquired from one batch of CFSs (n = 3).

	B. breve **DSM20091**	***B. breve*** **DSM20456**	***B. bifidum*** **DSM20082**	***B. bifidum*** **DSM20213**
L. crispatus **BC8-CFS** -7 h	190.52 ± 4.56	220.34 ± 10.21	180.38 ± 6.78	223.28 ± 23.6
-13 h	158.89 ± 6.21	187.35 ± 9.45	157.94 ± 4.56	190.12 ± 8.95
-24 h	103.43 ± 3.47	145.61 ± 7.23	102.63 ± 6.25	139.46 ± 6.32
L. gasseri **BC11-CFS** -7 h	212.32 ± 7.45	247.79 ± 9.64	209.52 ± 9.36	267.82 ± 8.91
-13 h	171.44 ± 8.12	215.58 ± 11.32	170.44 ± 7.45	231.33 ± 10.35
-24 h	124.51 ± 6.98	155.87 ± 8.74	123.59 ± 4.65	156.62 ± 6.98
L. vaginalis **BC17-CFS** -7 h	396.79 ± 11.25	326.91 ± 9.36	308.54 ± 10.87	355.72 ± 12.54
-13 h	313.58 ± 9.78	298.28 ± 10.14	214.32 ± 11.34	289.78 ± 13.24
-24 h	162.12 ± 6.52	189.29 ± 9.21	144.91 ± 7.12	201.23 ± 10.84
	B. bifidum **DSM20215**	***B. longum*** **DSM20219**	***B. infantis*** **DSM20088**	***B. infantis*** **DSM20090**
L. crispatus **BC8-CFS** -7 h	231.31 ± 9.78	168.88 ± 8.23	158.92 ± 3.36	210.04 ± 10.84
-13 h	195.32 ± 8.54	142.62 ± 7.54	132.58 ± 4.56	121.34 ± 6.65
-24 h	150.19 ± 6.32	99.52 ± 6.98	97.52 ± 3.58	98.48 ± 4.32
L. gasseri **BC11-CFS** -7 h	252.28 ± 11.24	184.54 ± 8.94	169.82 ± 7.65	250.12 ± 11.48
-13 h	225.27 ± 12.28	158.28 ± 5.28	145.75 ± 6.42	136.88 ± 6.65
-24 h	165.47 ± 8.98	125.57 ± 7.29	113.16 ± 6.94	112.47 ± 8.32
L. vaginalis **BC17-CFS** -7 h	318.77 ± 13.41	352.04 ± 11.32	232.92 ± 9.45	285.58 ± 12.34
-13 h	280.18 ± 11.47	225.64 ± 10.89	171.31 ± 8.36	156.03 ± 6.98
-24 h	215.49 ± 9.84	130.51 ± 8.54	125.74 ± 4.75	152.02 ± 5.65
	B. adolescentis **DSM20083**	***B. adolescentis*** **DSM20086**	***B. angulatum*** **DSM20098**	***E. coli*** **SO107**
L. crispatus **BC8-CFS** -7 h	230.61 ± 10.84	182.47 ± 7.21	220.14 ± 11.79	101.36 ± 6.21
-13 h	142.34 ± 6.74	161.29 ± 5.65	132.55 ± 5.45	95.39 ± 5.66
-24 h	128.46 ± 2.34	105.56 ± 9.12	118.33 ± 6.32	94.12 ± 4.57
L. gasseri **BC11-CFS** -7 h	270.42 ± 7.98	214.57 ± 7.48	259.29 ± 12.32	102.34 ± 2.23
-13 h	159.13 ± 4.36	175.76 ± 5.46	148.87 ± 6.33	99.09 ± 4.68
-24 h	132.39 ± 3.56	126.87 ± 3.77	124.51 ± 4.56	99.91 ± 8.99
L. vaginalis **BC17-CFS** -7 h	388.91 ± 8.99	300.04 ± 9.08	360.91 ± 7.89	101.47 ± 9.21
-13 h	321.54 ± 4.65	232.91 ± 10.17	300.34 ± 13.32	100.22 ± 4.56
-24 h	215.61 ± 11.32	143.53 ± 6.34	162.72 ± 5.45	98.18 ± 7.28

Table S4 Concentrations of metabolites identified in *L. vaginalis* BC17-CFS-7 h, BC17-CFS-13 h and BC17-CFS-24 h by means of ^{1}H-NMR. Concentrations are calculated as differences from MRS broth (mmol/L).

	**BC17-CFS-7 h**	**BC17-CFS-13 h**	**BC17-CFS-24 h**
Acetate	-0.08385	1.890963	3.462663
Acetone	0.003683	0.012908	-0.04407
Alanine	0.547327	0.583526	0.658338
4-Aminobutyrate	-7.917620	-7.699690	-7.705340
Ascorbate	-0.016450	-0.073410	-0.151510
Asparagine	0.079621	0.121927	0.139162
Aspartate	0.069302	0.076849	0.140977
2,3-Butanediol	0.140458	0.297788	0.333282
Carnitine	0.005071	-0.00128	-0.00976
Choline	-0.078930	0.010291	0.017831
Creatine	-0.01178	-0.04201	-0.06848
Creatinine	0.027159	0.040846	0.015137
Cysteine	0.244673	0.114412	0.142582
Cytidine	0.001450	0.007524	0
1,3-Dihydroxyacetone	0.0127420	0.015433	0.043588
Ethanol	32.15973	89.99636	119.7147
Formate	0.026622	0.166477	0.252582
Fructose	-1.522160	-2.413920	-2.498410
Glucose	-32.4321	-81.8033	-135.02
Glutamate	2.574019	3.274563	4.404634
Glycerol	0.386180	0.428580	0.698908
Glycine	0.046360	0.094093	0.153861
2-Hydroxyisovalerate	0.014838	-0.01615	-0.04721
4-Hydroxyphenylacetate	0.342421	0.050658	0.055779
4-Hydroxyphenyllactate	0.069730	0.010706	1.645295
Hypoxanthine	-0.01044	0.025296	0.051736
Inosine	-0.004840	0	0
Isobutyrate	-0.012030	-0.010950	-0.014350
Isoleucine	0.270583	0.332132	0.408987
Lactate	0.672027	2.423465	14.0042
Leucine	1.398733	1.217750	0.681371
Lysine	0.153855	0.196822	0.455287
Maltose	-0.468280	-1.439140	-1.439140
Mannose	-0.027060	-0.130870	-0.207690
Methanol	0.032158	0.054368	0.042424
Methionine	0.049827	0.053229	0.090273
N-Acetylaspartate	-0.004930	0.257167	0.654957
Galactose	-0.472420	-0.472420	-0.472420
Glutamine	0.002855	0.211727	0.180485
O-Acetylcholine	-0.178320	-0.479910	-0.513260
O-Phosphocholine	0.014631	0.036034	0.020396

Ornithine	1.266772	1.123998	0.826663
Phenylalanine	-0.073280	-0.210870	-0.244130
Proline	0.070373	0.001167	0.054416
Pyruvate	-0.120170	-0.122970	-0.112390
Ribose	-0.127440	-0.220100	0
Succinate	0.073251	0.112591	0.253292
Threonine	-0.164560	0.027505	0.151545
Thymine	0.021640	0.022490	0.020184
Trehalose	-0.120970	0.122650	-0.097190
Trigonelline	0.010096	0.002862	-0.016050
Tryptophan	0.064192	0.046386	0.009169
Tyrosine	0.329040	0.293650	0.238328
Uridine	-0.001300	0.0270603	-0.016440
Valine	0.483745	0.565348	0.812885
